# Survival of an HLA-mismatched, bioengineered RPE implant in dry age-related macular degeneration

**DOI:** 10.1016/j.stemcr.2022.01.001

**Published:** 2022-02-03

**Authors:** Amir H. Kashani, Jane S. Lebkowski, David R. Hinton, Danhong Zhu, Mohamed A. Faynus, Sanford Chen, Firas M. Rahhal, Robert L. Avery, Hani Salehi-Had, Clement Chan, Neal Palejwala, April Ingram, Wei Dang, Chih-Min Lin, Debbie Mitra, Juan Carlos Martinez-Camarillo, Jeff Bailey, Cassidy Arnold, Britney O. Pennington, Narsing Rao, Lincoln V. Johnson, Dennis O. Clegg, Mark S. Humayun

**Affiliations:** 1 Wilmer Eye Institute, Johns Hopkins University, 600 N. Wolfe Street, Baltimore, MD 21087 USA; 2 Regenerative Patch Technologies, 150 Gabarda Way, Portola Valley, CA 94028, USA; 3 Department of Pathology, Keck School of Medicine, University of Southern California, 1441 Eastlake Avenue, 7503, Los Angeles, CA 90033, USA; 4 Center for Stem Cell Biology and Engineering, Neuroscience Research Institute, Mail Code 5060, University of California, Santa Barbara, CA 93016, USA; 5 Orange County Retina Medical Group, 1200 N. Tustin Avenue, Suite 140, Santa Ana, CA 92705, USA; 6 Retina-Vitreous Associates Medical Group, 9001 Wilshire Boulevard, Suite 301, Beverly Hills, CA 90211, USA; 7 California Retina Consultants, 525 E. Micheltorena Street, Santa Barbara, CA 93103, USA; 8 Retina Associates of Southern California, 7777 Edinger Avenue, Suite 234, Huntington Beach, CA 92647, USA; 9 Southern California Desert Retina Consultants, University Park, 36-949 Cook Street, Suite 101, Palm Desert, CA 92211, USA; 10 Retinal Consultants of Arizona, 15401 North 29th Avenue, Phoenix, AZ 85053, USA; 11 Center for Biomedicine and Genetics, Beckman Research Institute of City of Hope, 1500 East Duarte Road, Duarte, CA 91010, USA; 12 USC Roski Eye Institute, USC Ginsburg Institute for Biomedical Therapeutics and Department of Ophthalmology, Keck School of Medicine, University of Southern California, 1450 San Pablo, Los Angeles, CA 90033, USA; 13 Department of Biomedical Engineering, Denney Research Center (DRB) 140, University of Southern California, 1042 Downey Way, Los Angeles, CA 90089, USA

**Keywords:** implant, retinal pigmented epithelium, parylene, clinical trial, histology, geographic atrophy, macular degeneration, allogeneic, stem cells, polarized

## Abstract

Cell-based therapies face challenges, including poor cell survival, immune rejection, and integration into pathologic tissue. We conducted an open-label phase 1/2a clinical trial to assess the safety and preliminary efficacy of a subretinal implant consisting of a polarized monolayer of allogeneic human embryonic stem cell-derived retinal pigmented epithelium (RPE) cells in subjects with geographic atrophy (GA) secondary to dry age-related macular degeneration. Postmortem histology from one subject with very advanced disease shows the presence of donor RPE cells 2 years after implantation by immunoreactivity for RPE65 and donor-specific human leukocyte antigen (HLA) class I molecules. Markers of RPE cell polarity and phagocytosis suggest donor RPE function. Further histologic examination demonstrated CD34^+^ structures beneath the implant and CD4^+^, CD68^+^, and FoxP3^+^ cells in the tissue. Despite significant donor-host HLA mismatch, no clinical signs of retinitis, vitreitis, vasculitis, choroiditis, or serologic immune response were detected in the deceased subject or any other subject in the study. Subretinally implanted, HLA-mismatched donor RPE cells survive, express functional markers, and do not elicit clinically detectable intraocular inflammation or serologic immune responses even without long-term immunosuppression.

## Highlights

• Clinical trial of allogeneic RPE cell transplant as AMD therapeutic
• Postmortem histology shows 2-year survival and function of donor RPE cells
• Transplanted RPE cells are mature, polarized, and phagocytic
• Serologic immune and clinical cellular inflammatory responses are not detected

## Teaser

In this article, Humayun and colleagues report the histologic assessment of a bioengineered RPE cell implant, called CPCB-RPE1, in a clinical trial for advanced dry age-related macular degeneration. The data show that highly HLA-mismatched allogeneic stem cell-derived RPE cells implanted subretinally survive and maintain a mature phenotype in the subretinal space for >2 years.

## Introduction

Non-neovascular age-related macular degeneration (NNAMD) is a major unmet medical need that affects millions of people in the Western world (Kashani, 2016; Miller, 2013; Nazari et al., 2015). Vision loss in NNAMD is highly correlated with loss of the retinal pigmented epithelium (RPE) in a pattern of geographic atrophy (GA) (Lambert et al., 2016; Miller, 2013). Macular translocation surgery (Benner et al., 2002; Cahill et al., 2005), transplantation of autologous adult RPE cells (Binder et al., 2004; Peyman et al., 1991; van Meurs and Van Den Biesen, 2003), and injection of suspensions of human embryonic stem cell (hESC)-derived RPE (hESC-RPEs) cells (Schwartz et al., 2012, 2015, 2016) have been pursued as potential treatments for NNAMD. Use of induced pluripotent stem or hESC-RPE cells in a different but related disease, neovascular age-related macular degeneration, has also been studied (Mandai et al., 2017; da Cruz et al., 2018). However, the long-term safety, survival, function, and immunogenicity of ocular transplantation in NNAMD remain incompletely characterized.

We have conducted a phase 1/2a clinical trial using a composite implant (CPCB-RPE1) consisting of a monolayer of hESC-RPE cells cultured on a microfabricated parylene membrane as a replacement for atrophic RPE in subjects with advance GA where the treated eye was legally blind or worse (best corrected visual acuity ≤ 20/200). The CPCB-RPE1 subretinal implant is designed to cover the majority of the macula, measuring 3.5 × 6.25 × 0.006 mm, and has a monolayer of approximately 100,000 RPE cells. We provide data showing that the allogeneic CPCB-RPE1 implant does not elicit intraocular inflammation or an acute rejection response, and that functional donor RPE cells survive within the area of host GA at least 2 years after implantation. These observations provide evidence for long-term survival, function, and limited immunogenicity of allogeneic hESC-RPE cells implanted subretinally into a human eye.

## Results

Fifteen subjects were enrolled in a phase 1/2a clinical trial and implanted with CPCB-RPE1 (see experimental procedures for a detailed description of the trial design and subjects recruited). The median age of the cohort was 78 (range 69–85) years, with nine and six subjects being female and male, respectively (Table 1). The CPCB-RPE1 implant has two key components: an ultrathin parylene membrane, which serves as the substrate onto which the second component, RPE cells derived from pluripotent stem cells, attach and polarize. The RPE cells are allogeneic, being derived from a single hESC line, and no attempt was made to match human leukocyte antigen (HLA) class I or class II alleles between the donor RPE cells on the implant and the recipient. All implanted subjects had >50% of 16 tested HLA class I and II alleles mismatched with donor RPE (Table 1). Subjects received a short course of immunosuppression consisting of 0.075 mg/kg/day tacrolimus (Astellas Pharma US, Northbrook, IL, USA) from day −8 to day 42 to achieve a serum trough range of 3–10 ng/mL. At day 42, tacrolimus doses were tapered by half every week until day 60, when immunosuppression was terminated.

**Table 1:** Summary of subject demographics and summary of allele mismatches at three MHC class I and five MHC class II alleles

Subject ID	Age	Sex	No. of mismatched HLA alleles with allogeneic implanted RPE cells
204	85	F	9 of 12
125	84	F	14 of 16
303	84	M	11 of 16
128	69	F	9 of 16
304	82	M	10 of 16
305	69	M	12 of 16
501	78	F	13 of 16
130	78	F	11 of 16
401	78	F	≥9 of 16
403	80	F	12 of 16
216	77	F	12 of 16
404	73	M	13 of 16
606	70	M	13 of 16
502	77	M	13 of 16
607	76	F	12 of 16

HLA molecular typing was performed on both alleles of the HLA-A, HLA-B, HLA-C, HLA-DRB1, HLA-DQB1, HLA-DQA1, HLA-DPB1, and HLA-DPA1 loci. F, female; M, male.

### Clinical course and gross pathology of subject 125 at 2 years after CPCB-RPE1 implantation

**Figure 1:**
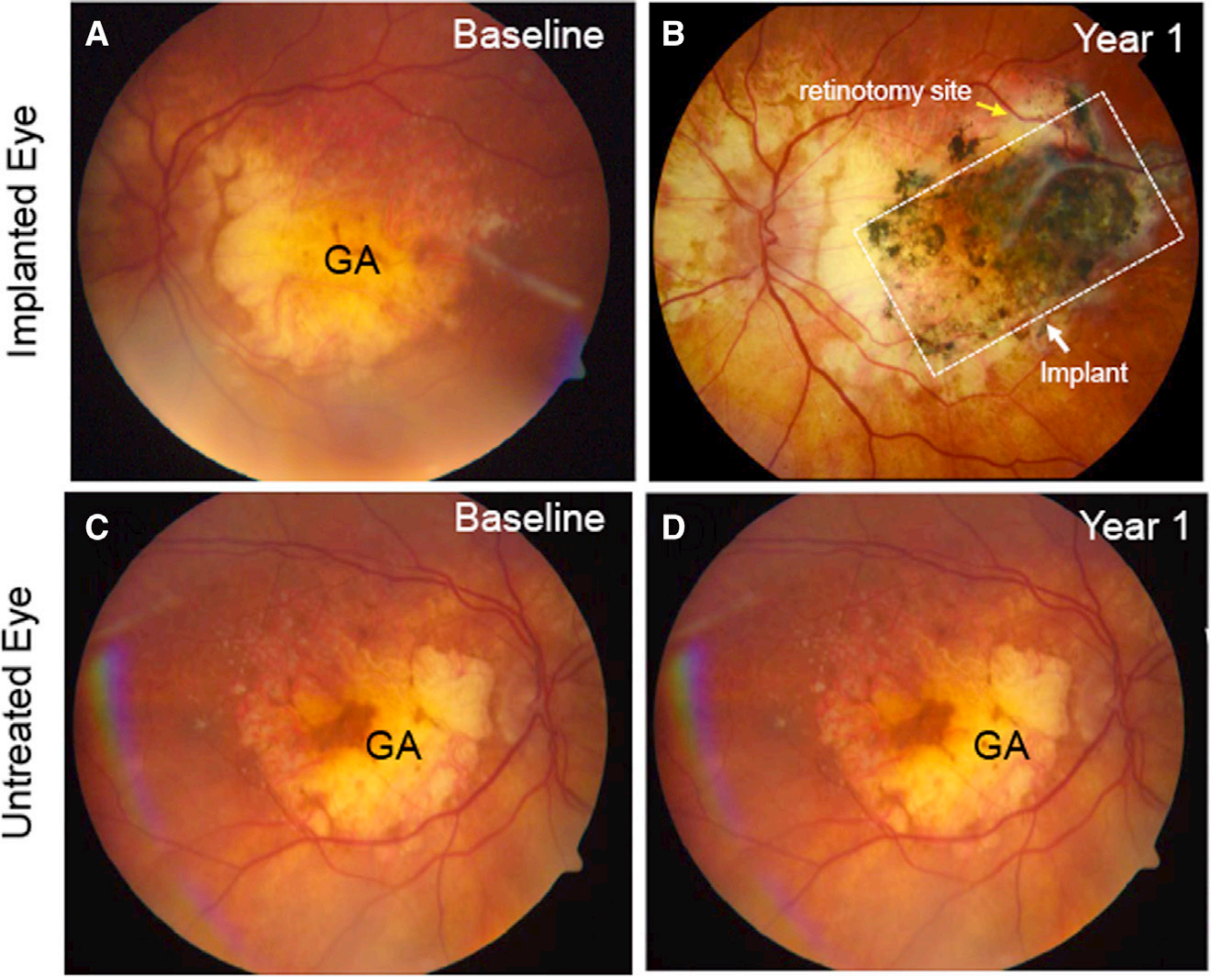
Color fundus photographs of subject 125 at baseline and 1 year after CPCB-RPE1 surgical implantation into the subretinal space (A) Preoperative photograph demonstrates variable areas of depigmentation in the central macula consistent with geographic atrophy (GA) in advanced dry age-related macular degeneration. (B) Postoperative fundus photographs of the same region at 1 year after CPCB-RPE1 implantation demonstrates the presence of the implant and its associated pigmented cells covering a large portion of the GA lesion. One edge of the pigmented implant is denoted by a white arrow for reference. The retinotomy site is denoted by a yellow arrow. (C) Fundus photograph of the nonimplanted eye at baseline. (D) Fundus photograph of the nonimplanted eye 1 year later.

Subject 125 was an 84-year-old woman who passed away from pneumonia approximately 2 years after CPCB-RPE1 implantation in the left eye; the cause of death was unrelated to the study procedures or implant. Preoperative evaluations in this subject demonstrated a very large area of GA (46.4 mm^2^) and count fingers visual acuity in the implanted eye. In contrast, best-corrected visual acuity in the nonimplanted eye was 20/50, and GA area was 37.0 mm^2^. Although there was variation in the pigment intensity, the implant remained pigmented throughout the follow-up period consistent with survival of donor RPE. Visual acuity in the implanted eye was unchanged at the 1- and 2- year follow-up visits. Visual acuity in the nonimplanted eye was unchanged from baseline at 1 year and decreased by three letters at 2 years (from 65 ETDRS [Early Treatment Diabetic Retinopathy Study] letters to 62 letters). There was no evidence of mass lesions or other unexpected anatomic abnormalities. Figure 1 provides fundus photo images of the implanted (Figures 1A and 1B) and nonimplanted eye (Figures 1C and 1D) from subject 125 at baseline and 1 year post-implantation. HLA molecular typing analysis of this subject in comparison with the H9 cell line-derived donor RPE cells on the CPCB-RPE1 implant demonstrated mismatch of 14 of the 16 class I and II alleles examined (Tables 1 and 2). Postmortem collection of the implanted and untreated eye was performed, and the samples were examined histologically for retinal structure, as well as implant RPE cell survival, phenotype, and function.

**Table 2:** HLA molecular typing analysis from subject 125 receiving CPCB-RPE1 and the donor H9 hESC line

HLA locus	Subject 125		H9 cell line source of CPCB-RPE1	
	Allele 1	Allele 2	Allele 1	Allele 2
A	01:01:01	11:01:01	02:01:01	03:01:01
B	07:02:01	35:01:01	35:03:01	44:27:01
C	04:01:01^a^	07:02:01	04:01:01	07:04:01
DRB1	04:07:01	11:01:01	15:01:01	16:01:01
DQB1	03:01:01	03:01:01	05:02:01	06:02:01
DQA1	03:03:01	05:05:01	01:02:01	01:02:02
DPB1	02:01:02	02:01:02	04:01:01	10:01:01
DPA1	01:03:01^a^	01:03:01	01:03:01	02:01:01
No. of mismatched alleles with H9	14/16			

a Alleles are a match with an HLA allele expressed in H9 cells.

### RPE survival and function at 2 years in subject 125

**Figure 2:**
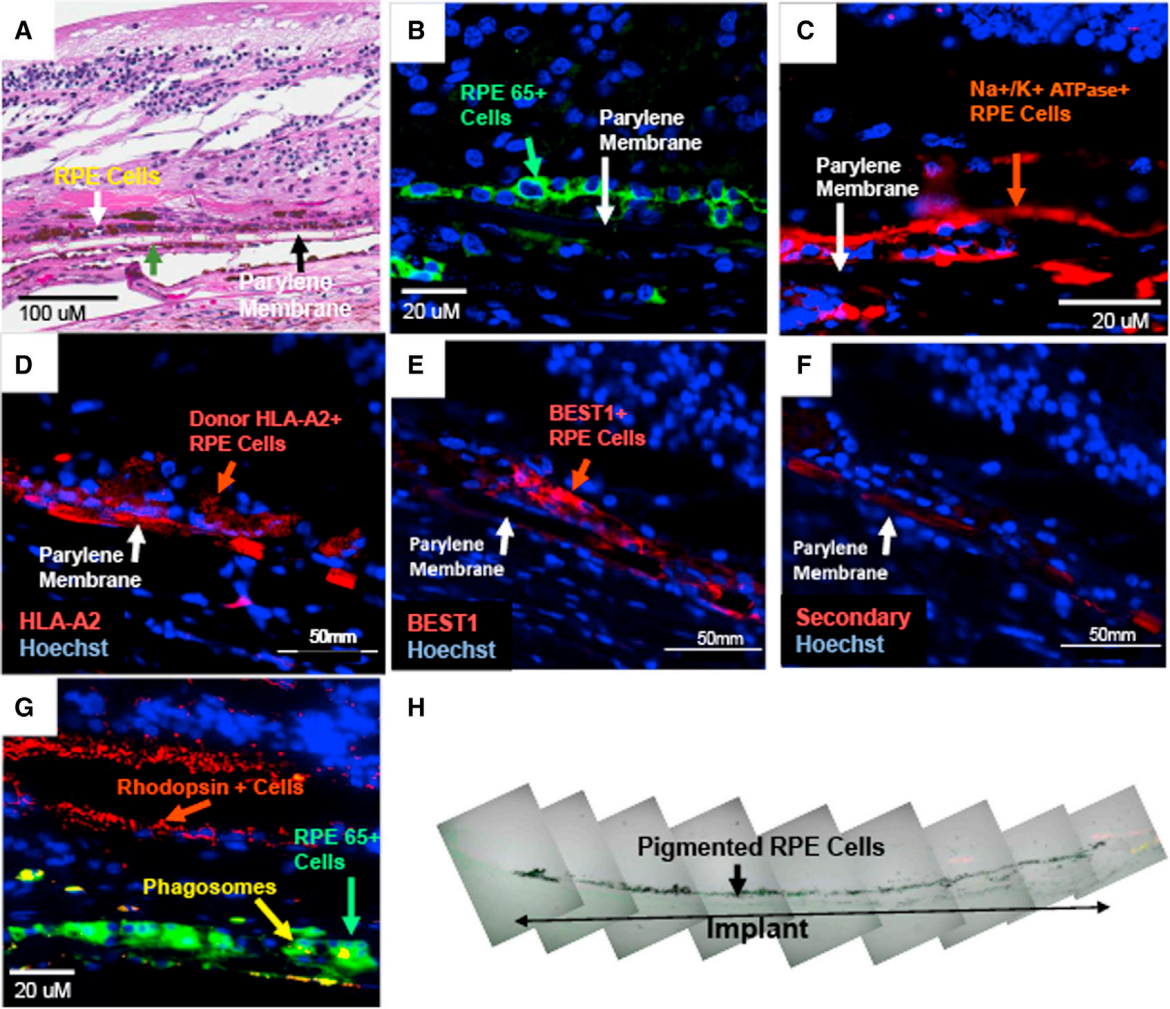
Retinal histopathology in subject 125 at 2 years post-implantation shows RPE survival and phagocytotic activity (A) H&E staining of implanted retina. The parylene membrane (black arrow) that forms the basement membrane-like scaffold for the RPE cells appears as a translucent rectangular object with alternating thin (6 μm) and ultrathin (0.4 μm) regions on H&E images but is not directly visible in subsequent fluorescence images. H&E staining within the area of the implant demonstrated a monolayer of pigmented RPE cells associated with the parylene membrane; RPE cells also were occasionally observed to be associated with the underside of the membrane (green arrow) as a result of growth of RPE cells around the edge of the membrane onto the bottom surface during implant production. The retina overlying the implant exhibits severe disorganization of outer retinal layers consistent with geographic atrophy. (B) Immunofluorescence for RPE65 (green) is present on the implant RPE cells. (C) Immunofluorescence of a similar region demonstrates that donor RPE cells express Na^+^/K^+^-ATPase (red) in a largely apical distribution consistent with mature and functional RPE. (D–F) Immunohistochemical identification of donor RPE. (D) Human leukocyte antigen serotype A2 (HLA-A2) immunoreactivity (red) in donor RPE cells closely associated with the parylene membrane (arrowhead). The HLA-A2 serotype is specifically expressed by donor, but not recipient, cells. Fluorescence associated with the parylene membrane is a consequence of the Superboost staining procedure and non-specific binding of the Tyramide solution. (E) Immunostaining for bestrophin (BEST1, red) in an adjacent section to that shown in (D) confirms the identity of cells associated with the parylene membrane (arrowhead) as RPE cells. (F) Secondary antibody control shows only artifactual staining associated with the parylene membrane (arrowhead). (G) Yellow immunofluorescence represents red-stained phagosomes (rhodopsin) in green-stained cytoplasm (RPE65) within the donor RPE of the implant in small granules suggestive of the presence of phagosomes containing photoreceptor outer segments. Rhodopsin staining, normally associated with rod photoreceptors, is present in outer segment-like rosette structures in the overlying atrophic retina. (H) Phase-contrast image of implant area showing the pigmented RPE cells along the entire length of the CPCB-RPE1 implant. Blue fluorescence in (B)–(D) indicates DAPI staining of cell nuclei.

H&E staining within the area of the implant demonstrated a monolayer of pigmented RPE cells associated with the parylene membrane in all available sections (Figure 2A); pigmented cells also were occasionally observed associated with the underside of membrane. Preclinical *in vitro* studies have shown that the donor RPE cells occasionally grow around the rim of the implant onto its bottom surface during manufacture and can persist there after experimental implantation in rats (data not shown). The RPE cells on the implant were immunoreactive for RPE65 and Na^+^/K^+^-ATPase, which are proteins essential for normal RPE function (Figures 2B and 2C). RPE65 is a visual cycle protein that participates in the conversion of all-*trans* retinol from overlying photoreceptor cells to 11-*cis* retinol in RPE cells (Kiser and Palczewski, 2010; Schachat et al., 2018). Apical localization of Na^+^/K^+^-ATPase is characteristic of RPE polarization, a feature of mature RPE *in vivo* (Schachat et al., 2018). The donor origin of the implant-associated RPE cells in subject 125 was confirmed by positive immunostaining for the HLA class I antigen, HLA-A2, which is expressed by donor cells (Figure 2D), but not by those of subject 125. Cells similarly positioned on the parylene membrane stained with antibodies to bestrophin, a cytosolic calcium-activated ion channel found primarily on RPE cells (Figure 2E), while staining with the secondary antibody alone showed only weak background staining of the parylene membrane (Figure 2F). The RPE cells on the implant did not stain for the recipient-specific HLA-B7 antigen (Figures S1A and S1B), although HLA-B7^+^ cells could be observed particularly in the choroid. There was no evidence of cell proliferation in implant-associated cells as assessed by Ki67 immunoreactivity (Figures S1C and S1D). Preclinical studies had documented staining of donor RPE cells by the HLA-A2 antibody (Figures S2A and S2B) in implanted rats. Staining of RPE cells in the non-treated eye of subject 125 by the HLA-B7 antibody (Figures S2C and S2D) was confirmed in parallel analyses.

Photoreceptor nuclei were not detected in the area of the implant; however, focal areas of rhodopsin staining associated with photoreceptor-like structures in rosette-like configurations within the area of GA and immediately above the CPCB-RPE1 implant were noted (Figure 2G). In addition, within the RPE65 positive RPE cells, yellow (red and green co-positive) inclusions were observed likely representing rhodopsin-positive phagosomes in the RPE cells and suggestive of the possible functionality of the implanted RPE cells (Figure 2G). Composite images across the entire length of the implant (Figure 2H) indicate that pigmented cells can be found along the full extent of the implant.

### Immune cell infiltrates in subject 125

**Figure 3:**
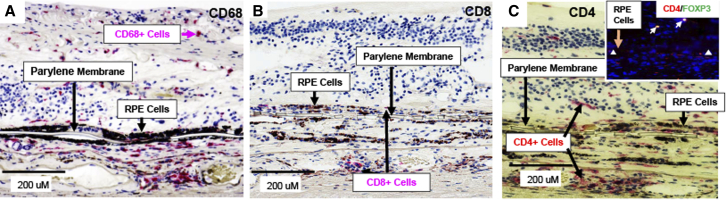
Retinal histopathology of cellular immune responses in subject 125 at 2 years post-implantation (A–C) The parylene membrane that forms the basement membrane-like scaffold for the RPE cells appears as a translucent rectangular object with alternating thin (6 μm) and ultrathin (0.4 μm) regions. All sections were stained with hematoxylin and counterstained as follows: (A) CD68 (red), a marker of macrophages, is present in the retina and choroid; (B) CD8 (red), a marker of cytotoxic T lymphocytes, also is observed occasionally in the choroid and in the retina near the implant; (C) CD4 (red), a marker of Th lymphocytes, is distributed throughout the retina and choroid and fluorescence imaging of double-labeled CD4^+^ and FOXP3^+^ cells (red-rimmed cells with yellow nuclei) found in the retina (inset). DAPI (blue) was used as a counterstain to label nuclei in the inset in (C).

Immunohistochemistry was performed for the macrophage marker, CD68, and the T cell markers CD8 and CD4 in both the implanted and non-implanted eyes. CD68^+^ cells were more abundant and more widely distributed in the retina and choroid of the implanted eye (Figure 3A), which had a much larger area of GA compared with that in the less severely affected, non-implanted eye (Figure S3B). The distribution of CD68^+^ cells in the implanted eye was throughout the retina and choroid, with higher concentrations being found in the choroid. There were infrequent CD8^+^ cytotoxic T cells in the choroid and adjacent to the implant in the treated eye (Figure 3B), with CD8^+^ cells being particularly concentrated in the area adjacent to the Bruch's membrane in the untreated eye (Figure S3C). CD4^+^ Th cells were also found in the retina and choroid surrounding the implant at 2 years (Figure 3C) and were also prevalent in the choroid of the non-implanted eye (Figure S3D). Some of the CD4^+^ cells in the retina of the implanted eye were also positive for FOXP3 (Figure 3C, inset) potentially indicative of a regulatory or immune-suppressive effect of these cells.

### Histopathology assessment in subject 125

**Figure 4:**
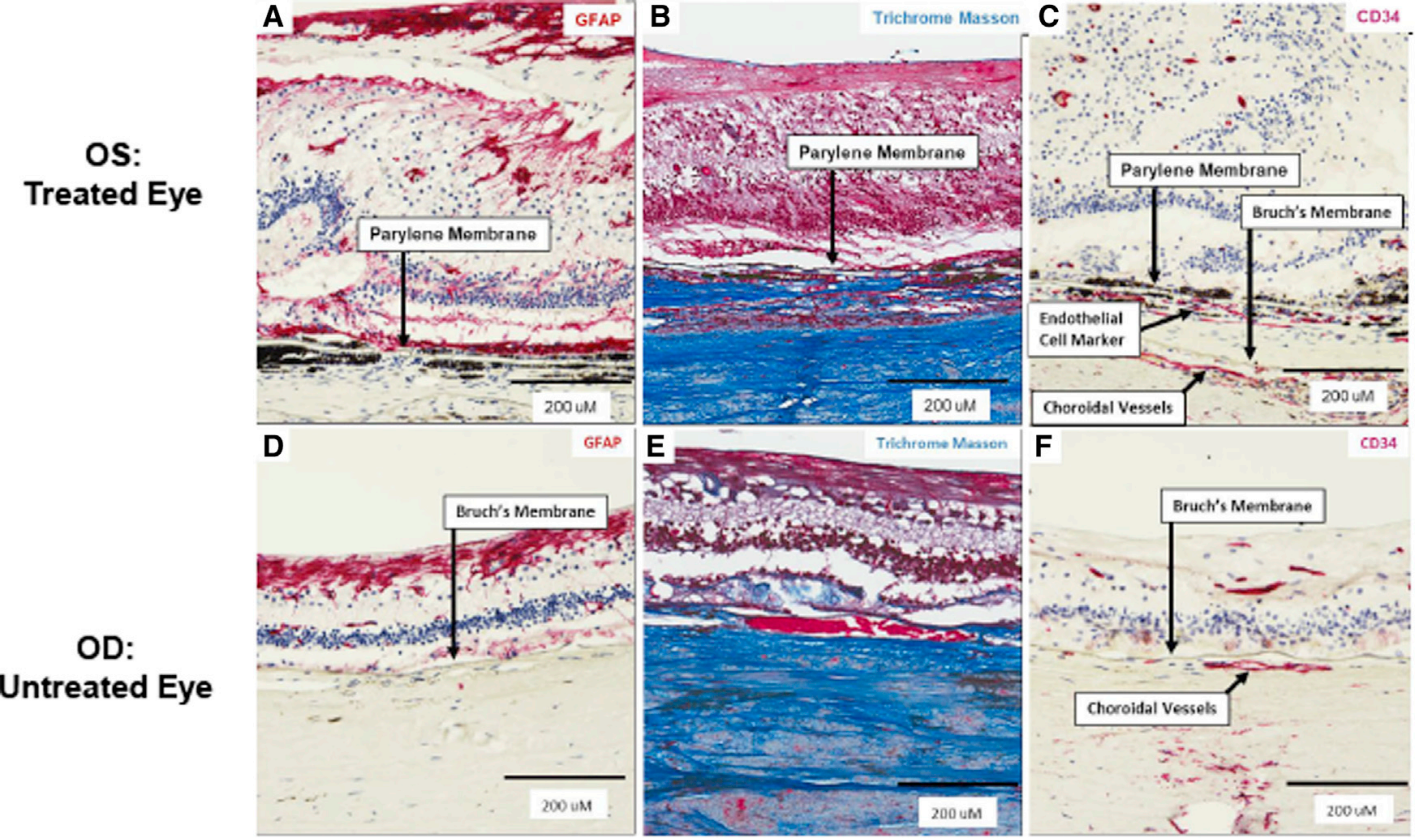
Histopathology of intraretinal gliosis and sub-implant material in subject 125 at 2 years post-implantation The parylene membrane that forms the basement membrane-like scaffold for the RPE cells appears as a translucent rectangular object with alternating thin (6 μm) and ultrathin (0.4 μm) sections. (A) Staining for glial fibrillary acidic protein (GFAP, red) demonstrates diffuse gliosis in the retina, but no staining in the subimplant space. (B) Trichrome Masson staining demonstrates staining of collagen (blue) beneath the implant that is consistent with the staining of the native choroid below it. (C) CD34, an endothelial cell marker (red) associated with vascular-like structures, is present in the sub-implant tissue immediately adjacent to the implant. There is also staining of native choroidal vessels beneath Bruch's membrane. Bottom row: gliosis in the area of geographic atrophy in the non-implanted, contralateral control eye of subject 125 with severe, advanced dry age-related macular degeneration. (D) GFAP (red) counterstained with hematoxylin demonstrates diffuse gliosis of the retina. (E) Trichrome Masson staining in area of geographic atrophy. (F) CD34, a marker of endothelial cells (red), counterstained with hematoxylin demonstrates staining of intraretinal and choroidal vessels; no staining is observed in the subretinal space. (A, C, D, and F) Counterstained with hematoxylin (blue) to identify cell nuclei.

There was intraretinal glial fibrillary acidic protein (GFAP) staining diffusely in the area of GA in both the implanted (Figure 4A) and non-implanted (Figure 4D) eye indicating gliosis. Although there was GFAP^+^ staining overlying the CPCB-RPE1 implant, there was no GFAP positivity observed anywhere below the entire length of the CPCB-RPE1 implant (Figure 4A). Masson Trichrome staining demonstrated collagen throughout the sub-implant space, which was homogeneous in color, cellularity, and tissue organization to the scleral collagen (Figure 4B). Immunostaining for CD34, an endothelial cell marker, demonstrated vascular-like channels containing red blood cells immediately below the implant in the subretinal space and separated from choroidal vasculature by Bruch's membrane (Figure 4C). Histopathology of the area of GA in the contralateral, non-implanted eye did not demonstrate any subretinal vascular structures (Figure 4F).

### Immunologic assessments of study cohort

Despite the lack of HLA class I or II matching and the use of only short-term immunosuppression, multiple clinical assessments throughout the first year of follow-up did not reveal evidence of intraocular inflammation, including cell, flare, vascular staining, inflammatory infiltrate, retinitis, vitreitis, vasculitis, or choroiditis in any subject in the study.

To determine whether subjects in the clinical trial developed humoral immune responses to donor-specific HLA antigens on the implant, the presence of antibodies to specific HLA class I and II antigens was monitored on serial peripheral blood samples collected from 13 patients at baseline and 90, 180, and 365 days post-implantation by an independent laboratory (University of California Los Angeles [UCLA] Immunogenetics Center) (see experimental procedures). The assay employed detects the presence of antibodies to 97 HLA class I and 99 HLA class II molecules, including those present on the donor RPE cells. One subject (1/13 or 7.6%) had pre-existing antibodies to a single-donor HLA antigen (Table 3), while six (6/13 or 46.1%) subjects had pre-existing antibodies to non-donor HLA molecules at baseline (Table 3). These pre-existing antibodies to specific HLA molecules remained detectable at the majority of follow-up time points (Table 3). Twelve (12/13 or 92.3%) subjects never developed detectable antibodies to any donor HLA antigen through 1 year post-implantation of CPCB-RPE1. Only one subject had developed “weak” antibody response to a single HLA class II molecule expressed by donor RPE cells (DQB1) at 180 and 365 days post-implant (Table 3). The results indicate that the implanted subjects did not develop robust humoral immune responses to the mismatched HLA class I or II molecules present on the donor cells of the implant.

**Table 3:** Longitudinal analysis of antibodies to donor HLA antigens on RPE cells of CPCB-RPE1

Subject	No. of mismatched subject HLA alleles with CPCB-RPE1	Detection of antibodies to donor HLA antigens			
		Baseline	Day 90 of follow-up	Day 180 of follow-up	Day 365 of follow-up
128	9 of 16	−	–	not done	–
303	11 of 16	−a	−a	not done	−a
304	10 of 16	−	–	not done	–
305	12 of 16	−	–	–	–
130	11 of 16	−	–	–	–
501	13 of 16	−a	−a	−a	−a
401	13 of 16	−a	−a	−a	−a
216	12 of 16	−a	−a	–	–
403	12 of 16	−	−	–	–
404	13 of 16	−a	−	+^a^ (weak Ab to donor DQB1)	+^a^ (weak Ab to donor DQB1)
606	13 of 16	−	−	−	−a
502^b^	13 of 16	+^a^ (moderate Ab to donor DQB1)	+^a^ (moderate Ab to donor DQB1)	+^a^ (moderate Ab to donor DQB1)	+^a^ (moderate Ab to donor DQB1)
607	12 of 16	−	−a	−a	−a

Minus signs (−) indicate no antibodies to donor HLA antigens detected; plus signs (+) indicate antibodies to donor HLA antigens detected. Mean fluorescence intensity (MFI) was used to classify the antibodies as not present, weak, moderate, or strong. The definitions of those classifications were: (1) not present, MFI < 1,000; (2) weak, MFI 1,000–3,000; (3) moderate, MFI 3,000–5,000; and (4) strong, MFI > 5,000. It should be noted that such analysis was not performed for most time points for subject 125, because this subject was early in the trial at a time when this assay was not available. A flow-based panel reactive antibody (PRA) test was performed on baseline and day 90 samples from this patient. The results from the PRA assay indicated that subject 125 had a low level of weak antibodies to HLA class I and no antibodies to HLA class II molecules at both baseline and day 90. This was confirmed using the bead-based assay at the 365 days of follow-up.

a Subject had antibodies to non-donor HLA molecules, the identity of which were consistent across time points tested. The majority (61%) of these were characterized as weak binding antibodies, with 26% classified as moderate and 13% classified as strong.

b It is of interest that subject 502, who had pre-existing antibodies to donor HLA antigen DQB1, showed survival of the RPE cells as assessed by fundus photography.

## Discussion

There are several challenges and questions surrounding therapeutic cell replacement strategies, such as validating a configuration for cell replacement (cell suspension versus 3D structure), developing feasible delivery methods, assuring long-term donor cell survival, mitigating allogeneic immune responses, and confirming function of donor cells in pathologic host tissue (Kashani, 2016; Nazari et al., 2015). Our results from a phase 1/2A study indicate sustained survival of allogeneic RPE cells in the subretinal space, and a lack of clinical ocular inflammation upon use of a short-term immunosuppression protocol despite delivery of an HLA-mismatched RPE cell implant in subjects with highly advanced NNAMD. Most importantly, these 2-year data demonstrate that this implant and procedure did not result in any potentially catastrophic outcomes, including migration of the implant into the vitreous, aggressive neovascularization and proliferative vitreoretinopathy, posterior uveitis, or any process that could jeopardize the safety of the subject.

Although the lack of clinically detectable inflammation does not eliminate the possibility of any immune response, the histopathologic evidence of donor RPE survival, polarization, and likely phagocytotic function 2 years post-implantation do not support immune-mediated rejection of the allogeneic cells. Due to the extreme severity of GA in this subject, it is also not surprising that there was no improvement in vision, but the histopathologic and clinical persistence of RPE cells suggests that a therapeutic effect might be possible in less advanced disease. Clinical examination of all remaining subjects in the ongoing study (Kashani et al., 2021) also demonstrates persistent pigmentation of the implant through 1 year, supporting the histologic findings in the one subject presented here.

The unique histopathologic data from this study provide hypothesis-generating observations that are invaluable for further investigation. Staining for GFAP demonstrates diffuse gliosis in the implanted and non-implanted retina but no staining in the sub-implant space. Trichrome Masson staining demonstrates collagen deposition beneath the implant that is consistent with the staining of the native choroid below it. The stark difference in the histopathology above and below the implant demonstrates a lack of gliotic encapsulation. Similarly, the presence of CD34^+^ vascular structures immediately subjacent to the implant suggests that donor RPE may elicit formation of a highly localized vascular supply to support the graft function, possibly through the action of vascular endothelial growth factor, a known secretory product of RPE cells. The absence of persistent clinically evident hemorrhage or choroidal neovascularization and the survival of the overlying RPE during the 2-year period suggest that this is not necessarily a pathologic response. Several recent studies demonstrate that the presence of “asymptomatic macular neovascularization” or “quiescent macular neovascularization” is not uncommon and may play a protective effect in terms of hindering progression of GA (Laiginhas et al., 2020). Immunohistochemistry also demonstrates the presence of rhodopsin (i.e., rod photoreceptor opsin) in the retina overlying the implant, suggesting persistent rhodopsin expression in neurosensory retinal tissue in an area of long-standing GA.

Some animal models including non-human primates would have predicted immune rejection of allogeneic RPE cell introduced into the subretinal space (McGill et al., 2018; Sohn et al., 2015). However, mature, polarized monolayers of allogeneic fetal RPE and hESC-RPE have been shown to avoid immune rejection when transplanted into ocular and non-ocular sites (Idelson et al., 2018; Keino et al., 2018; Wenkel and Streilein, 2000). This controversy has led some to use autologous, induced pluripotent stem cell-derived RPE cells (Mandai et al., 2017). Successful pharmacologic immunosuppression has also been demonstrated to be effective in clinical trials but with significant risk in the elderly population (Schwartz et al., 2015; da Cruz et al., 2018). In our study, ophthalmoscopic examination demonstrated no evidence of inflammation in any of the 15 implanted subjects, and there was no evidence of humoral immunity throughout the first year as measured in peripheral blood. The absence of inflammation on clinical examination is supported by the histopathologic data from the current study, which shows that a highly mismatched, donor RPE monolayer survived 2 years after implantation in a highly degenerate retina with only a 60-day postoperative immunosuppression regimen with low-dose tacrolimus. The relatively low number of CD8^+^ cytotoxic T cells in the area of the implant and the presence of CD4^+^/FOXP3^+^ cells in the retina may contribute to survival of the highly mismatched RPE cells or at least not their destruction.

There are several possible factors specific to our study that may explain these findings. As mentioned above, monolayers of mature, polarized RPE, such as that of the CPCB-RPE1 implant, demonstrate enhanced survival (Brant Fernandes et al., 2016; Diniz et al., 2013) and immune tolerance (Keino et al., 2018; Wenkel and Streilein, 2000). Additional factors that likely made significant contributions to RPE survival in this study include: (1) the use of a parylene scaffold, which is a US pharmacopeia class VI biocompatible material (highest biocompatibility for materials) (Stark, 1996); (2) a surgical approach that minimizes the retinal incision size (Kashani et al., 2020); and (3) implantation in subjects with NNAMD in which the blood retinal barrier is less compromised than in active neovascular AMD (Algvere et al., 1997; Schultz et al., 2019). The short-term immunosuppression regimen used in this clinical trial may also impact the long-term survival of the RPE cells by providing protection during the peri-implantation period when inflammatory responses might be maximal. Collectively, these observations show that subretinal implantation of mature, polarized, and confluent RPE, such as the CPCB-RPE1, may not require an HLA-matched donor RPE in an immunocompetent human host (e.g., subject 125). These findings can inform the clinical trial design and choice of donor RPE cells in future cell-based ocular therapies for GA associated with NNAMD. The generalizability of these findings to other diseases, such as neovascular AMD, and other donor cell types, such as photoreceptors, will require additional investigation.

## Experimental procedures

### Study design

The study design (Kashani et al., 2018) and surgical methods (Kashani et al., 2020) have been described previously in detail. Institutional Review Board approval was obtained from the University of Southern California, as well as the Western Institutional Review Board for other participating sites. Informed consent was obtained from all subjects. Clearance of an Investigational New Drug application (IND) was obtained from the Food and Drug Administration for a prospective, non-randomized, single-arm, interventional phase 1/2a study to recruit and enroll up to 20 subjects to assess the safety and potential efficacy of the investigational implant called California Project to Cure Blindness Retinal Pigment Epithelium (CPCB-RPE1). A data monitoring and safety committee provided independent oversight of the study and reviewed all results and adverse events. The primary outcome measure of the study was safety, as assessed by multiple clinical examinations up to 365 days after implantation. The stopping rules for the study were: (1) development of an expanding mass, (2) accelerated loss of visual acuity in the implanted eye, (3) enucleation of the eye, and (4) failure of implant delivery. The preliminary results of the first five enrolled subjects were published (Kashani et al., 2018), and the detailed surgical methods and perioperative surgical results were also published (Kashani et al., 2020).

The CPCB-RPE1 implant has two key components: an ultrathin parylene membrane that serves as the substrate onto which the second component, RPE cells derived from pluripotent stem cells, can attach and polarize. Specifically, the CPCB-RPE1 implant is 3.5 × 6.25 × 0.006 mm in dimension and consists of a monolayer of approximately 100,000 mature, polarized, and pigmented hESC-RPE cells on the parylene substrate (Koss et al., 2016; Stark, 1996). The RPE cells are allogeneic, and no attempt was made to match HLA class I or II alleles between the donor RPE cells on the implant and the recipient. CPCB-RPE1 was manufactured under cGMP (City of Hope, Duarte, CA, USA) and supplied to the surgical site (University of Southern California, Los Angeles, CA, USA).

### Study subjects

Inclusion criteria for subjects were previously described (Kashani et al., 2018) and consisted of subjects 55–85 years of age with advanced NNAMD, GA, pseudophakia, and severe vision loss. Subjects with a history of any other vision-threatening disease, including neovascular age-related macular degeneration or health conditions that would prevent general anesthesia, were excluded from the study. Other key exclusion criteria include history of malignancy within the previous 5 years, history of enrollment in another clinical trial within the previous 3 months, history of active or untreated infectious disease, or any history of immunosuppression or immune dysfunction. Detailed enrollment criteria are available at ClinicalTrials.gov: NCT02590692.

### CPCB-RPE1 surgery and immunosuppression

Details of the surgical implantation procedure have been previously described in detail, and video illustrations of the surgery are also available (Kashani et al., 2020; Koss et al., 2016). In brief, subjects underwent outpatient surgery for subretinal implantation of a single CPCB-RPE1 on study day 0 using a 23-gauge pars plana vitrectomy approach. Insertion of the CPCB-RPE1 implant was performed with an experimental injector that was designed to fold and deliver the implant to the subretinal space through a small retinotomy (Kashani et al., 2020; Koss et al., 2016). Each enrolled subject received immunosuppression using 0.075 mg/kg/day tacrolimus (Astellas Pharma US, Northbrook, IL, USA) from day −8 to day 42 to achieve a serum trough range of 3–10 ng/ml. Subsequent to day 42, doses were tapered by half every week until day 60 when immunosuppression was terminated. Subjects received a single intravenous injection of 250 mg methylprednisolone sodium succinate (SOLU-MEDROL; Pfizer) prior to surgery on day 0.

### Postoperative clinical evaluations and retinal imaging

The presence or absence of retinal findings was assessed by the site principal investigators using standard clinical evaluations, color fundus photographs, and optical coherence tomography (OCT) imaging.

### Histopathology, immunohistochemistry, and immunofluorescence

One subject died of causes unrelated to the study 2 years after surgical implantation of CPCB-RPE1. Gross evaluation of both enucleated eyes was performed by an expert ocular pathologist (N.R.). Serial sections (7 μm) of both eyes were obtained through the entire macula for analyses, including hematoxylin and eosin (H&E), immunofluorescence, and histochemistry (Table S1). Masson-Trichrome staining was performed for visualization of collagen. For immunostaining, paraffin-embedded sections were deparaffinized using serial sections washed in xylene and rehydrated with descending ethanol rinses. Deparaffinized samples were subject to heat-induced antigen retrieval using citrate buffer (pH 8.0) and pressure cooker set to maximum pressure for 3 min. Samples were subsequently incubated with 3% hydrogen peroxide to quench endogenous peroxidase activity. Samples were stained with primary antibodies and in some cases use of the standard Superboost Alexa Fluor 594 Tyramide Reagent (B40957; Thermo Fisher) protocol.

### HLA genotyping and immunologic assessments

All subjects in the trial and the H9 hESC line that was the source material for RPE cell differentiation for CPCB-RPE1 were genotyped for alleles at three HLA class I loci and five HLA class II loci using molecular typing analysis (UCLA Immunogenetics Center, Los Angeles, CA). In addition, blood samples were obtained from all subjects prior to CPCB-RPE1 implantation, as well as post-implantation (days 90, 180, and 365) for assessment of humoral immune responses to the allogeneic donor RPE cells. For this assessment, a fluorescence-based bead assay (One Lambda LABScreen) that can detect serum antibodies to individual HLA class I and II antigens of H9 (donor) and non-H9 (recipient) origin was completed for 13 subjects. This latter assay is referred to as the “single HLA antigen-antibody test” and was also performed at the UCLA Immunogenetics Center. The single HLA antigen-antibody assay can detect antibodies to 97 HLA class I and 99 HLA class II molecules, including all of the donor HLA antigens.

## Author contributions

Conceptual design, collection/assembly of data, data analysis and interpretation, manuscript writing, and final manuscript approval, A.H.K., J.S.L., M.S.H., and D.R.H.; collection/assembly of data and final manuscript approval, F.M.R., R.L.A., H.S.-H., S.C., C.C., D.Z., and M.A.F.; data analysis and interpretation, manuscript writing, and final manuscript approval, L.V.J.; provision of study material and final approval of manuscript, W.D., C.-M.L., B.O.P., C.A., and J.B.; collection and assembly of data and final approval of manuscript, J.C.M.-C. and D.M.; collection and assembly of data, manuscript writing, and final approval of manuscript, A.I.; collection and assembly of data, data analysis and interpretation, and final approval of manuscript, N.R.; data analysis and interpretation and final approval of manuscript, J.S.L, M.S.H, D.R.H., D.O.C.; financial support, provision of study material, data analysis and interpretation, and final approval of manuscript.

## Conflicts of interests

The University of Southern California, D.O.C., D.R.H., M.S.H., L.V.J., and J.S.L. have financial interests in the subject matter of this study. D.O.C., D.R.H., and M.S.H. have an equity interest in and are consultants for Regenerative Patch Technologies (RPT). J.S.L., L.V.J., J.B., C.A., M.A.F., and B.O.P. are employees of RPT. A.H.K. receives speaking fees, grants, and honoraria from Carl Zeiss Meditec AG, unrelated to the topic of this study. The technology described in this publication is covered by the following issued US patents related to the parylene membrane and implant (US 8,808,687 and 10,188,769 submitted by the University of Southern California, the California Institute of Technology, and the Regents of the University of California with inventors including M.S.H., D.O.C., L.V.J., and D.R.H. and US 8,877,489 submitted by the California Institute of Technology and the University of Southern California with inventors including M.S.H.; the RPE cells (US 9,850,463 and 10,246,682 submitted by the Regents of the University of California and the University of Southern California with inventors including D.O.C., L.V.J., and D.R.H.), and US 9,458,428 (submitted by the Regents of the University of California with inventors that include D.O.C. and B.O.P.). RPT holds exclusive license to these patents. The other authors declare no competing interests.

## Data Availability

There are no accession numbers or genetic information relevant to this study. Requests for materials should be directed to Regenerative Patch Technologies (J. Lebkowski; jane@ regenerativepatch.com) and will be supplied upon completion of a material transfer agreement, which will contain a description of the proposed research using the materials.

## Supplemental information

Document S1. Figures S1–S3 and Table S1

Document S2. Article plus supplemental information
