# Supplementary material for: Survival of an HLA-mismatched, bioengineered RPE implant in dry age-related macular degeneration
Source: Stem Cell Reports. 2022 Feb 3;17(3):448–58. doi: 10.1016/j.stemcr.2022.01.001 (PMC9039755; doi:10.1016/j.stemcr.2022.01.001)
Supplement: Document S1. Figures S1–S3 and Table S1 [file mmc1.pdf]

Stem Cell Reports, Volume *17*

Supplemental Information

Survival of an HLA-mismatched, bioengineered RPE implant in dry age-related macular degeneration

Amir H. Kashani, Jane S. Lebkowski, David R. Hinton, Danhong Zhu, Mohamed A. Faynus, Sanford Chen, Firas M. Rahhal, Robert L. Avery, Hani Salehi-Had, Clement Chan, Neal Palejwala, April Ingram, Wei Dang, Chih-Min Lin, Debbie Mitra, Juan Carlos Martinez-Camarillo, Jeff Bailey, Cassidy Arnold, Britney O. Pennington, Narsing Rao, Lincoln V. Johnson, Dennis O. Clegg, and Mark S. Humayun

Figure S1

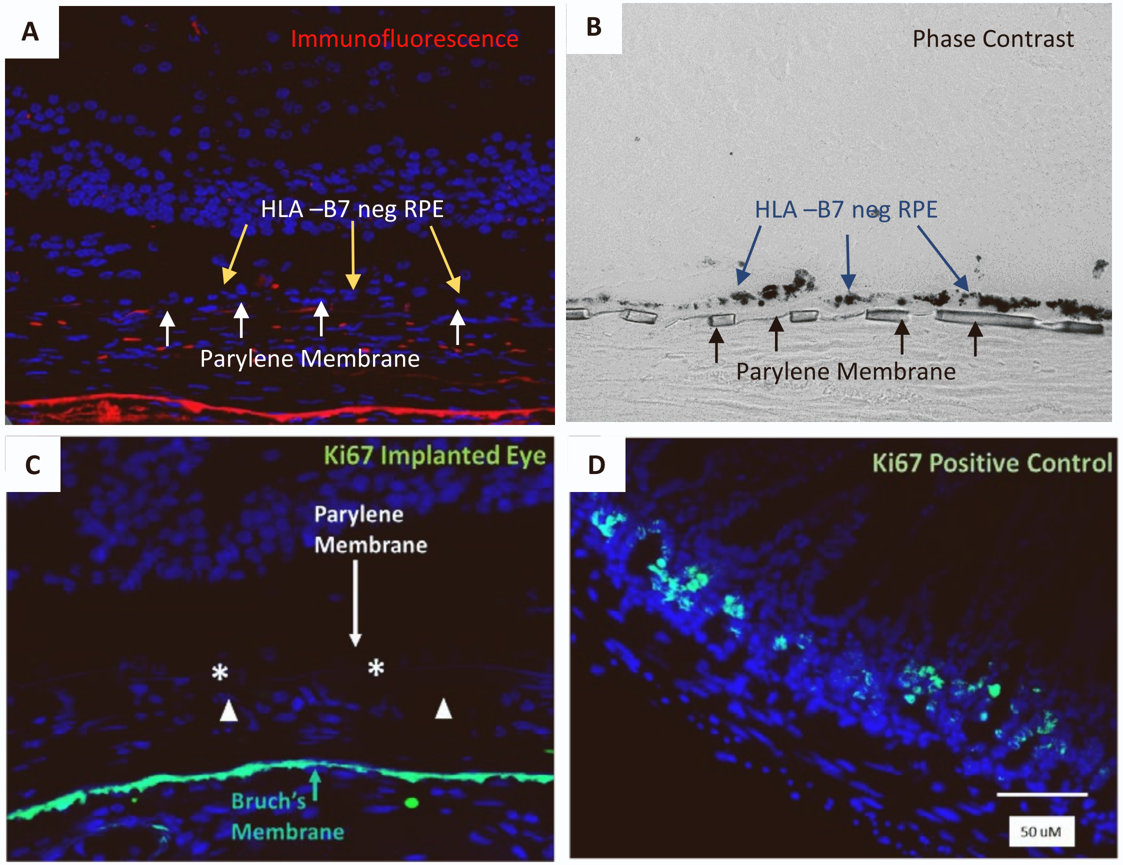
A
Immunofluorescence
HLA –B7 neg RPE
Parylene Membrane
B
Phase Contrast
HLA –B7 neg RPE
Parylene Membrane
C
Ki67 Implanted Eye
Parylene Membrane
Bruch's Membrane
D
Ki67 Positive Control
50 uM

Figure S2

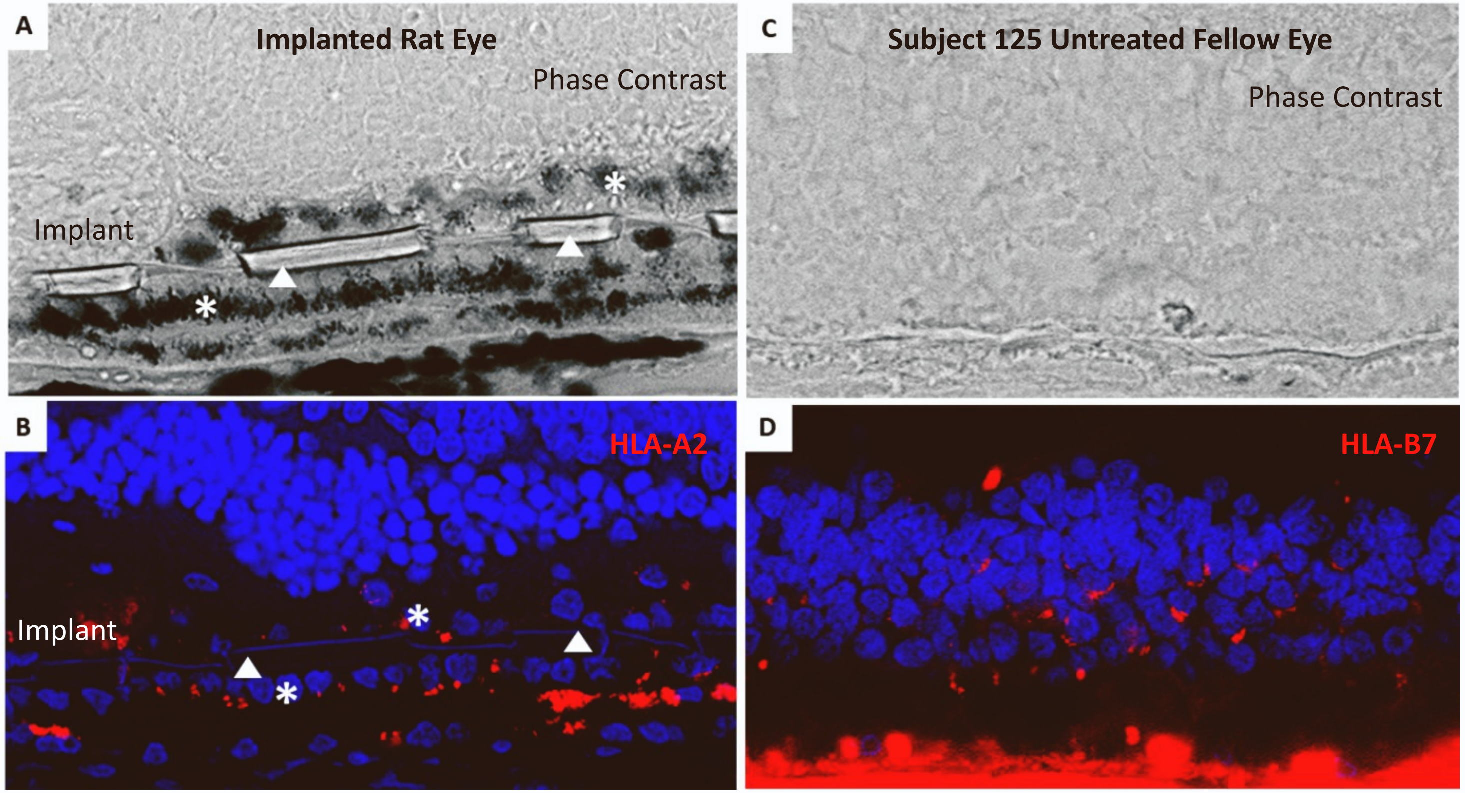
A
Implanted Rat Eye
Phase Contrast
Implant
B
HLA-A2
Implant
C
Subject 125 Untreated Fellow Eye
Phase Contrast
D
HLA-B7

Figure S3

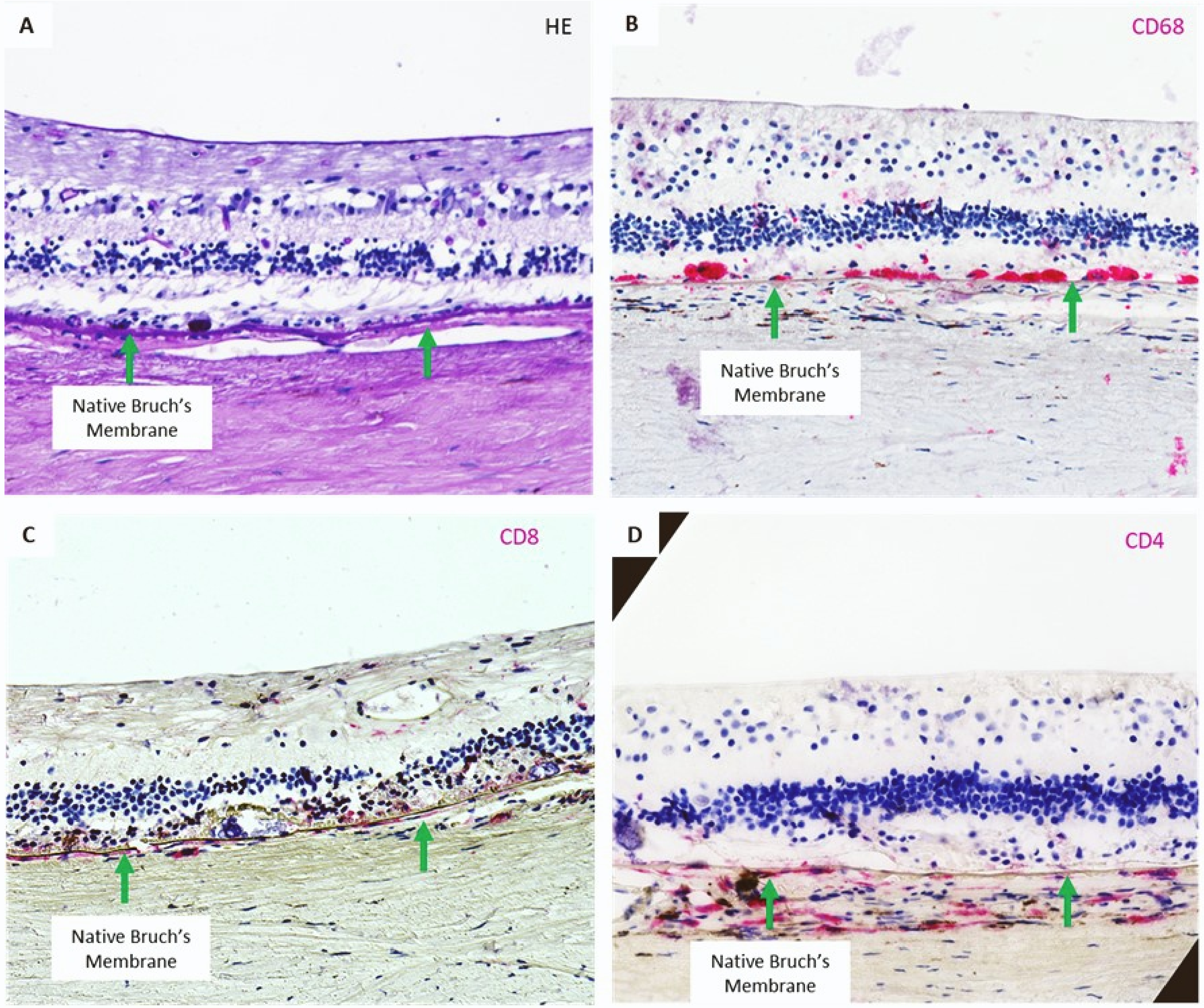
A
HE
Native Bruch's Membrane
B
CD68
Native Bruch's Membrane
C
CD8
Native Bruch's Membrane
D
CD4
Native Bruch's Membrane

Supplemental Figures Titles and Legends

Figure S1. Donor RPE Cells Do Not Express Host HLA Markers and are Non-Proliferative. Supplemental Data to Figure 2. Blue in A, C, D represents DAPI fluorescence associated with cell nuclei with immunostaining as follows: (A) HLA-B7 (red), a marker of the host cells, does not colocalize with donor RPE cells on CPCB-RPE1. HLA-B7 immunoreactivity underneath the implant corresponds to host tissue in sub-implant space. Note the fact that only a portion of the nucleated cells retina and choroid express HLA-B7 is not surprising as most non-regenerating cells of the retina either lack or have low expression of HLA molecules (30); (B) Phase contrast image of previous panel demonstrates location of donor RPE that are HLA-B7 negative and parylene membrane for reference. Note that the parylene membrane has alternating thick (labelled arrows) and thin sections. (C) There is no evidence of cell proliferation as assessed by Ki67 staining (green). The white triangles indicate the position of the parylene membrane and the asterisks, the position of the implant RPE cells. Green line is artifactual staining of Bruch's membrane below the implant. (D) Positive control for Ki67 antibody in mouse small intestine tissue.

Figure S2. Test Staining of human HLA-A2 and HLA-B7 antibodies. Supplemental Data to Figure 2. Controls for HLA-A2 and HLA-B7 antibody staining. A rat-sized CPCB-RPE1 implant containing the same HLA-A2+ RPE cells that were used for human clinical use were implanted into the subretinal space of the degenerating retina of the Royal College of Surgeons rat. The white arrow heads indicate the parylene membrane and the white asterisks denote the RPE cells on the implant. Note that the parylene membrane has alternating thick (arrows) and thin sections. (A) A phase contrast image of the pigmented RPE cells on the implant. (B) A

fluorescence image of the same field where the red human HLA-A2-specific antibody staining indicates the presence of the donor human HLA-A2+ RPE cells associated with the parylene membrane. (C) A phase contrast image of the non-treated eye in Subject 125. (D) A fluorescence image of the same field stained with a human specific HLA-B7 antibody which stains host cells within the retina. B and D are counterstained with DAPI (blue) to show cell nuclei.

Figure S3. Macrophages and Lymphocytes in the Non-implanted Retina of Subject 125. Supplemental Data to Figure 3. Immunohistochemistry from the non-implanted, contralateral control eye of Subject 125. All sections are from within the area of geographic atrophy. Green arrows indicate Bruch's membrane as a landmark for orientation. (A), Hematoxylin and eosin staining of the retina demonstrates complete loss of outer retinal features. (B), Hematoxylin-stained section with immunostain for a marker of macrophages, CD68 (red). (C), Hematoxylin-stained section with immunostain for a marker of cytotoxic T cells CD8, (red). (D), Hematoxylin-stained section with immunostain for a marker of helper T cells, monocytes, and macrophages, CD4 (red).

Supplemental Tables

Table S1. Summary of Antibodies Used

Antibody Target	Species	Antigen Location	Manufacturer	Catalog#	Dilution
Rhodopsin	Mouse	Rods and Phagocytosed Rod Outer Segments	Abcam	Ab3267	1:400
Ki67	Rabbit	Proliferating Cells	Abcam	Ab16667	1:500
GFAP	Rabbit	Glial Cells	Abcam	Ab7260	1:2000
Na/K ATPase	Mouse	Apical Side of Polarized RPE	ThermoFisher	MA1-16731	1:100
RPE65	Rabbit	RPE	Abcam	Ab105366	1:500
RPE65	Rabbit	RPE	Abcam	Ab231782	1:1000
BEST1	Mouse	RPE	Novus Biologicals	NB300-164	1:50
FOXP3	Mouse	Regulatory T Cells	Abcam	Ab20034	1:100
CD68	Mouse	Macrophages	Abcam	Ab31630	1:100
CD31	Mouse	Endothelial cells	Abcam	Ab9498	1:1000
CD34	Mouse	Hematopoetic and vascular tissue	Abcam	Ab54208	1:500
CD4	Mouse	Helper T Cells	Sigma/Millipore	AMAB90754	1:100
CD8	Mouse	Cytotoxic T cells	Leica Biosystems	4B11	neat
HLA-A2	Mouse	Cells expressing the class I HLA-A2 allele such as the CPCB-RPE1 implanted RPE cells	Lifespan Biosciences (LSBio)	LS-B10938-0.25	20µg/mL
HLA-B7	Mouse mAb IgG2b	Cells expressing the class I HLA-B7 allele such as observed in Subject 125	Abcam	Ab236491	1:100
Goat anti-mouse Ig G conjugated with Rhodamine	Goat	Goat affinity purified antibody to mouse IgG	Jackson ImmunoResearch	115-025-146	1:100
Goat anti-rabbit Ig G conjugated with FITC	Goat	Goat affinity purified antibody to rabbit IgG	Jackson ImmunoResearch	111-095-144	1:100
Goat anti-mouse Ig G conjugated with Rhodamine	Goat	Goat affinity purified antibody to mouse IgG	American Qualex	A106RS	1:50
Goat anti-rabbit IgG conjugated with FITC	Goat	Goat affinity purified antibody to rabbit IgG	American Qualex	A102FS	1:50
Tyramide Superboost Kit with Alexa Fluor 594	Goat	Goat affinity purified antibody to mouse IgG Poly HRP conjugated secondary antibody	Thermofisher	B40915	
